# A New Horizon in Reproductive Research with Pluripotent Stem Cells: Successful In Vitro Gametogenesis in Rodents, Its Application to Large Animals, and Future In Vitro Reconstitution of Reproductive Organs Such as “Uteroid” and “Oviductoid”

**DOI:** 10.3390/biology11070987

**Published:** 2022-06-29

**Authors:** Sho Yoshimatsu, Iori Kisu, Emi Qian, Toshiaki Noce

**Affiliations:** 1 Department of Stem Cell Biology and Medicine, Graduate School of Medical Sciences, Kyushu University, Higashi-ku, Fukuoka 812-8582, Japan; 2 Research Fellow of Japan Society for the Promotion of Science (JSPS), Chiyoda-ku, Tokyo 102-0083, Japan; 3 Department of Physiology, Keio University School of Medicine, Shinjuku-ku, Tokyo 160-8582, Japan; emisen@keio.jp; 4 Laboratory for Marmoset Neural Architecture, RIKEN Center for Brain Science, Wako-City 351-0198, Japan; toshiaki.noce@riken.jp; 5 Department of Obstetrics and Gynecology, Keio University School of Medicine, Shinjuku-ku, Tokyo 160-8582, Japan; iori71march@hotmail.co.jp

**Keywords:** pluripotent stem cell, germ cell, reproductive tract, large animal

## Abstract

**Simple Summary:**

Functional gametes, such as oocytes and spermatozoa, have been derived from rodent pluripotent stem cells, which can be applied to large animals and ultimately, to humans. In addition to summarizing these topics, we also review additional approaches for in vitro reconstitution of reproductive organs. This review illustrates intensive past efforts and future challenges on stem cell research for in vitro biogenesis in various mammalian models.

**Abstract:**

Recent success in derivation of functional gametes (oocytes and spermatozoa) from pluripotent stem cells (PSCs) of rodents has made it feasible for future application to large animals including endangered species and to ultimately humans. Here, we summarize backgrounds and recent studies on in vitro gametogenesis from rodent PSCs, and similar approaches using PSCs from large animals, including livestock, nonhuman primates (NHPs), and humans. We also describe additional developing approaches for in vitro reconstitution of reproductive organs, such as the ovary (ovarioid), testis (testisoid), and future challenges in the uterus (uteroid) and oviduct (oviductoid), all of which may be derived from PSCs. Once established, these in vitro systems may serve as a robust platform for elucidating the pathology of infertility-related disorders and ectopic pregnancy, principle of reproduction, and artificial biogenesis. Therefore, these possibilities, especially when using human cells, require consideration of ethical issues, and international agreements and guidelines need to be raised before opening “Pandora’s Box”.

## 1. Introduction

In all mammalian species, germ cells are pivotal for the transmission of genomic and epigenetic information to the next generation. In the beginning of life, one oocyte and spermatozoon fuse together to generate a zygote, which is totipotent and harbors the potential to form extraembryonic tissues and an embryo. After implantation, the embryo itself develops with the aid of the uterus and placenta until birth. The process of full-term embryonic development until birth (around 20 days in mice, 280 days in humans) is very complicated and not fully understood. In addition, the germ-cell linage arises in an embryo (epiblast) at the postimplantation stage and undergoes epigenetic reprogramming for acquisition of totipotency at the timing of fertilization [1,2]. Because these processes are highly important for understanding the principle of mammalian biology, significant efforts have been made using a variety of animal models and human samples. Here, based on the biological principle in vivo, we review backgrounds and recent advances in in vitro reconstitution of germ cells and reproductive organs, and discuss future potentials and ethical concerns.

## 2. Development of Germ Cells In Vivo

In mammals, primordial germ cells (PGCs), the origin of the germ-cell linage, are specified in the postimplantation embryo at the posterior gastrulating region (in rodents, E6.5 epiblast in mice [3]) or in the nascent amnion (in nonrodents including rabbits, pigs, and primates [4,5,6]). Owing to a small number of founder PGCs (~40 cells), they proliferate in the yolk sac until the migration to the embryonic gonad (genital ridge; E8.5–11.5 in mice) expressing late-PGC-specific markers, including VASA [7]. After the entry of the PGCs into the gonad, sex specification of PGCs occurs with the aid of embryonic gonadal somatic cells (GSCs) in the embryonic gonad (genital ridge), which express the *SRY* gene on chromosome Y in males and not in females (E12.5 in mice). During mitosis of PGCs in the yolk sac and gonad, they undergo epigenetic reprogramming, including erasure of parental imprinting and reinitiation of sex-specific imprints mainly on the differentially methylated regions [1]. After mitosis and sex specification, the PGCs are differentiated into precursor spermatogonial stem cells (pre-SSCs; in males) or oogonia (in females). Only pre-SSCs and further differentiated spermatogonial stem cells (SSCs; in postnatal males) retain the mitosis ability, which contributes to unlimited supply of spermatozoa in males in contrast to the limited number of oocytes in females. After birth, oogonia are differentiated into primary oocytes (at the prophase of meiosis I) with the aid of female GSCs (mainly by follicle cells) and stay in a dormant state until ovulation-related signals are received in puberty [8]. When an oocyte is ovulated, it reinitiates meiosis until the metaphase of meiosis II until fertilization. On the other hand, SSCs undergo asymmetric mitosis to produce primary spermatocytes. Mainly with the aid of Sertoli cells in testes, primary spermatocytes enter meiosis I to produce secondary spermatocytes, and then the secondary spermatocytes (1n, 2c) enter meiosis II to form spermatids (1n). Maturation of the haploid spermatids results in the drastic morphological transformation of spermatids to spermatozoa harboring an arrow-like unique cell shape, highly condensed nuclear DNA with protamine, and the potential of active movement with a flagellum. This overall process is called spermatogenesis.

## 3. Successful In Vitro Reconstitution of Mouse Gametes

Historically, since the allogenic chimera formation of mouse PSCs with germline contribution by injection to host blastocysts was achieved in the 1980s [9], researchers mainly relied on this technique for the production of gene-targeted mice until the 2010s, when the application of genome-editing technologies directly in mouse zygotes became available [10,11]. Accordingly, the necessity of germ-cell study in mammals had been considered as less serious for decades. However, even in other rodent species, including rats, intensive efforts were made for obtaining germline-competent PSCs, which was finally achieved in 2010 [12], and has never been reproducibly achieved in other mammalian species, including primates [13,14,15], indicating a growing need for the promotion of germ-cell study. Although the critical reason(s) of the difficulty in chimeric formation (by PSC injection into host blastocysts) in mammalian species except rodents remains not fully understood, many studies have pointed out the cell-autologous differences between naïve PSCs (comparable to preimplantation epiblast, which is a ground state for rodent PSCs) and primed ones (comparable to postimplantation epiblast, which is a ground stage for nonrodent mammalian PSCs, including human ones) and may serve as key for chimeric contribution and proper differentiation during in vivo development. For example, growth factors critical for the maintenance of pluripotency are different between naïve and primed state (LIF vs. bFGF/Activin A), and antiapoptotic signals in naïve PSCs (vs cell-autologous apoptotic signals in primed PSCs) for survival during in vivo development may play a pivotal role for the higher chimeric contribution potential. Please refer to a review by Nichols and Smith [16] for further description of the two pluripotent states.

Attempts for mammalian in vitro gametogenesis have begun in mice. In 2003, putative oocytes were derived by initial spontaneous differentiation from mouse embryonic stem cells (ESCs) and subsequent maturation culture [17,18]. However, even with intensive efforts, these putative oocytes could not contribute to generate offspring, which is the most stringent criterion for germ-cell function. Therefore, researchers inferred that a deeper understanding of the signaling principle of germ-cell differentiation was required for in vitro gametogenesis.

Two years later, Ohinata et al. discovered that the expression of *Blimp1* is critical for germ-cell specification, such as the emergence of PGCs, by analysis of *Blimp1*-deficient mice [19]. Subsequently, the research group deciphered the key signaling pathways of mouse PGC specification, including Bone morphogen protein 4 (BMP4) signal-induced upregulation of *Blimp1* and *Prdm14*. As a result, they succeeded in in vitro PGC induction from E6.0 (pluripotent) epiblast cells at the postimplantation stage by supplementation of defined cytokines such as BMP4, stem-cell factor (SCF), epidermal growth factor (EGF), and leukemia inhibitory factor (LIF) using transgenic mice harboring *Blimp1-mVenus* (BV) and *Stella* (a PGC and mature germ-cell marker)*-ECFP* or *-Venus* (SC or SV) reporters [20]. The initial PGC specifier *T* (*Brachyury*) in mice was subsequently identified [21] as an upregulated gene by Wnt signaling from posterior extraembryonic tissues during gastrulation. The expression of the *T* gene is also involved in the mesoderm specification [22], which shows its dual function for the specification of mesodermal and germ-cell fates in a pluripotent state in mice. In addition, as the naïve pluripotency of mouse ESCs (comparable to preimplantation epiblasts) was not favorable to direct PGC induction, researchers differentiated ESCs into epiblast-like cells (EpiLCs) by a short-term culture with Activin A (ActA) and basic fibroblast growth factor (bFGF), which are important cytokines for proliferation and maintenance of primed pluripotency in postimplantation epiblasts [16]. Based on these findings, Nakaki et al. demonstrated that forced expression of the key PGC specifiers (downstream of *T*) such as *Blimp1*, *Prdm14*, and *Tfap2c* in mouse EpiLCs could induce primordial germ-cell-like cells (PGCLCs) without cytokines for PGC induction [23].

In parallel, the research group succeeded in the derivation of functional gametes following the induction of PGCLCs with the defined cytokines from both male and female mouse PSCs such as ESCs and induced pluripotent stem cells (iPSCs) with BV/SC reporters through partial EpiLC induction. Maturation and meiosis of the male PGCLCs were achieved by transplantation into testes of neonatal mice to form fertile spermatids, which were used for fertilization by the intracytoplasmic sperm injection technique [24]. Additionally, female PGCLCs could form fertile oocytes by coculture (reaggregation) with gonadal somatic cells (GSCs) isolated from E12.5 female mouse embryos followed by transplantation into the ovarian bursa of female adult mice [25,26]. These milestone studies have paved the way for “in vitro gametogenesis from stem cells” in mammals.

Instead of in vivo transplantation, reaggregation of female PGCLCs with female GSCs and further maturation culture in sequential optimized conditions made it possible to form fertile oocytes [27]. On the other hand, reaggregation of male PGCLCs with male GSCs (isolated from E12.5 male mouse embryos) resulted in differentiation of SSC-like cells (SSCLCs) from PGCLCs. Transplantation of the SSCLCs into seminiferous tubules of testes in adult male mice formed fertile spermatids [28]. More recently, further incubation of SSCLCs in organ-cultured testes [29] in vitro formed fertile spermatids [30]. However, these approaches still required live cell resources (GSCs) from embryos, which may impose limitations on/upon future application to large animal models, and ultimately to humans.

As an alternative to the usage of E12.5 mouse embryos, female GSCs, which mainly originate from the coelomic epithelium derived from the ventral part of the media-lateral plate mesoderm (M-LPM) followed by epithelial-to-mesenchymal transition (EMT) [31,32,33], were also successfully differentiated from mouse ESCs by a stepwise differentiation method. Reaggregation of induced GSCs and PGCLCs (so called “ovarioid”) contributed to maturation and meiosis of PGCLCs and finally produced fertile oocytes [34]. Thus, the “embryo-free” and “stem cell alone” in vitro gametogenesis system was finally achieved in mice.

Furthermore, using similar approaches (in vivo transplantation methods) with the mouse studies [24,25], functional gametes were derived from rat PSCs [35,36].

**Figure 1:**
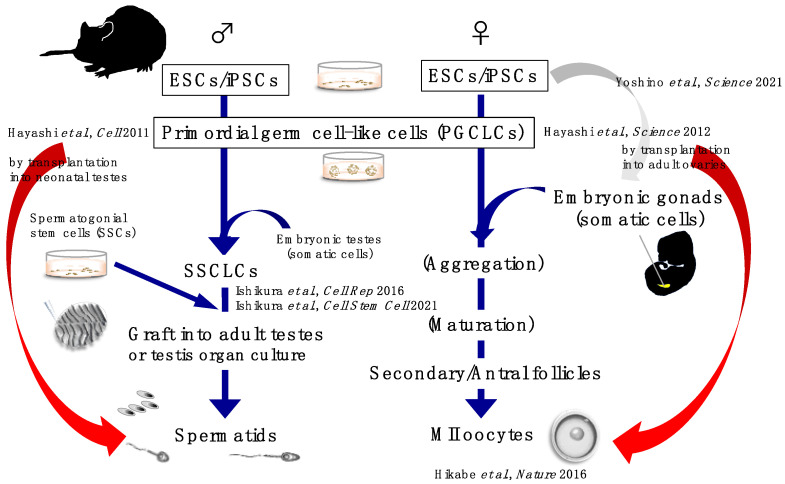
A graphical schematic of in vitro gametogenesis from murine PSCs.

We summarize in vitro gametogenesis from PSCs in mice in Figure 1.

## 4. Attempts of In Vitro Gametogenesis in Large Animals and Humans

**Figure 2:**
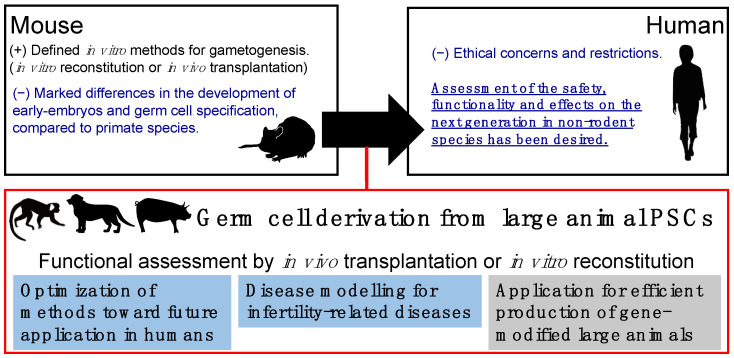
Significance of germ-cell study using large animal models. Cartoons of large animals (monkey, dog, and pig) were obtained from the graphical abstract of our previous study [40].

As described above, in vitro gametogenesis from rodent PSCs has been achieved in recent years. However, from a translational perspective, these approaches should be reproduced in large animals that are more closed to humans in size, physiology, and anatomy. The significance of large animal models in germ-cell study is summarized in Figure 2. In line with this necessity, we and other groups succeeded in the derivation of PGCLCs from PSCs of NHP species such as rhesus monkeys [37], cynomolgus monkeys [38,39], and common marmosets [40]. As nonrodent PGCLC derivation, porcine PSCs were also differentiated into PGCLCs [41].

Although the usage of human cells for germ-cell study is one of the most ethically restricted research topics, several groups have succeeded in the derivation of PGCLCs from human PSCs [38,42]. Furthermore, Yamashiro et al. demonstrated the maturation of human female PGCLCs to oogonia-like cells by long xenogenic reaggregation culture with GSCs from mouse E12.5 female embryos [43,44]. Similarly, Hwang et al. showed that human male PGCLCs were differentiated into pro-spermatogonia-like cells by long xenogenic reaggregation culture with GSCs from mouse E12.5 female embryos [45].

During the research progress with nonrodent PGCLC derivation, a variety of critical evolutionary differences between rodents and nonrodents were discovered, including the differences in the early PGC specifiers, nonrodent-specific critical functions of *EOMES* and *SOX17* [42,46] and downstream target genes of conserved PGC key markers such as *BLIMP1* and *PRDM14* [47], and indispensability of critical genes in rodent PGCs/PGCLCs including *T* and *SOX2* in those of nonrodents [48]. In addition, some evolutionary differences in the metabolism between rodent and primate GSCs, such as fatty-acid oxidization or carbohydrate metabolism, were also discovered [49]. Such issues may cause the “arrest” of differentiation toward functional gametes from PSCs in reported xenogenic culture methods as described above. Furthermore, Shami et al. performed single-cell analyses in mouse, monkey, and human testes, and discovered the unique but critical importance of *PIWIL4* for primate SSCs [50].

**Figure 3:**
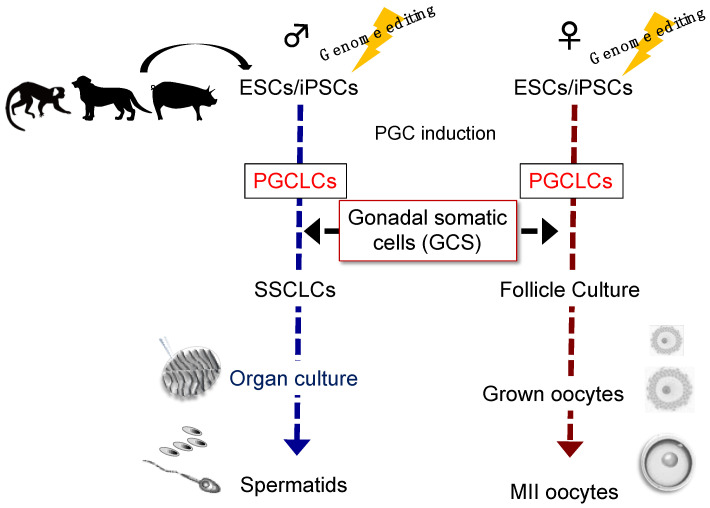
A graphical schematic of in vitro gametogenesis from large animal PSCs. Genome editing (introduction of PGC/mature germ cell reporters) is useful in ESCs/iPSCs for visualizing the differentiation. Cartoons of large animals (monkey, dog, and pig) were obtained from the graphical abstract of our previous study [40].

Since the usage of embryonic GSCs in large animals and humans is limited, the “ovarioid” method reported in mice as described above [34] and “testisoid” (named as GSC-reconstituted testis by adding the suffix -oid to testis) method in a coming study (unpublished) seem to harbor the current best potential for future in vitro gametogenesis using large animal PSCs, including those of NHPs (Supplementary Note). We summarize potential approaches for in vitro gametogenesis in large animals in Figure 3.

## 5. Female Reproductive Organs Required for Full-Term Development of an Embryo

Oviduct, also known as uterine tube or fallopian tube, is the place of fertilization in vivo in mammals. In particular, the “ampulla” of the oviduct, the upper and wider segment of the oviduct, compared to the lower and narrower segment of the oviduct “isthmus”, is the place that renders spermatozoa hyperactive to penetrate the extracellular oocyte coats (cumulus cells and zona pellucida). After fertilization, the zygote divides to form a morula (~32 cells; E2.5 in mice) in the isthmus of the oviduct. The morula develops to form a blastocyst (E3.5 in mice) harboring inner cell mass (future epiblast, hypoblast, and amnion) and surrounding trophectoderm (future part of placenta). The blastocyst hatches to break zona pellucida for implantation (E5.5 in mice). Implantation is taken place by attachment or intrusion of the hatched blastocyst to the uterine wall. Anatomical and mechanistical studies showed that there are great evolutionary differences in implantation machineries among mammalian species [51].

Last year, ex vivo (ex utero) development of mouse embryos was partially (from E5.5 to E11) achieved using an optimized three-dimensional (3D)-rotating whole-embryo culture condition [52]. This study paved the way for ex utero culture of mammalian embryos until the mid-gestation stage before embryos require the fetal–placental (fetoplacental) and maternal–placental (uteroplacental) blood-circulation systems for nutrition supply for further development [53]. Unsolved issues in Aguilera-Castrejon et al. are as following: (1) Implantation signals were not recapitulated; (2) the later part of the development (after E11 in mice) requires nutrition supply (by blood circulation in vivo); (3) an appropriate system to recapitulate the “birth” is required for achieving ex vivo full-term development.

On the other hand, many groups demonstrated the recapitulation of the implantation-like signal and postimplantation development of mammalian blastocysts upon the gastrulation stage using biocompatible gels and optimized culture methods [54,55,56,57,58,59], although the development of the embryos in these studies was limited upon the gastrulation and three-germ-layer segregation, which was far from the hindlimb formation stage (E11 in mice) as reported in the ex utero mouse study [52]. Thus, in vitro reconstitution of the uterus (“uteroid”, named as PSC-derived uterus-like organoid) from PSCs would make it feasible to achieve omnis ex vivo full-term embryonic development only using stem cells.

## 6. Potential Approaches for In Vitro Reconstitution of Female Reproductive Organs

Anatomically, the uterus harbors three layers, endometrium, myometrium, and perimetrium. Endometrium, the inner layer of the uterus, supports the implanted embryo’s development by forming embryonic/maternal chimeric placenta for nutrition supply by blood circulation and keeping a suitable environment for the embryo survival and development until birth [53]. Endometrium is mainly consisted of endometrial columnar epithelial cells and stromal cells that are responsive to the menstrual cycle, and reproduction-related hormones including estrogen and progesterone, although a recent single-cell study showed there are many more cell types in the human endometrium [60]. The middle layer, myometrium, is mainly composed of uterine smooth-muscle cells (uterine myocytes) that support the drastic size change of the uterus during gestation. The outer layer, perimetrium, is derived from the peritoneum surrounding the uterus fundus.

**Figure 4:**
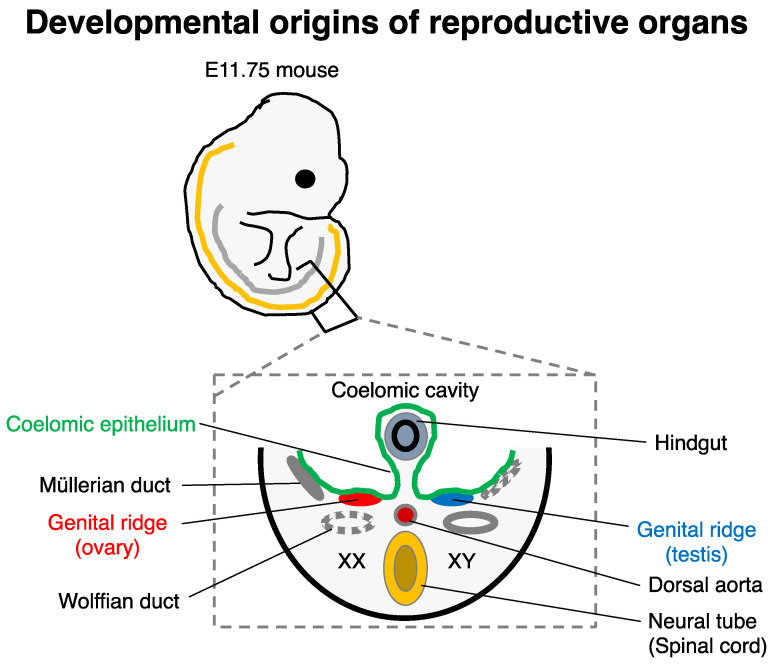
Developmental origins of reproductive organs. For example, E11.75 mice (**top**) is shown. The curved orange and gray lines show the spinal cord and the dorsal ridge of the coelomic epithelium. In a representative transverse section of the E11.75 mice (**bottom**), both female (XX; **left**) and male development (XY; **right**) are depicted.

Developmentally, the endometrium and part of myometrium are originated from the Müllerian ducts, also known as paramesonephric ducts. In a similar way to the genital ridge (capable of developing into the ovary in females), the Müllerian ducts (developed in E11.75–13.5 in mice [61]) are derived from the coelomic epithelium, and they also undergo invagination along the mesonephros [62,63], which is developmentally similar to that of the genital ridge (Figure 4). Part of the invaginating cells is converted to stroma by EMT. The rest of the coelomic epithelial cells are thought to form the peritoneum and pleura. During female embryogenesis, the absence of anti-Müllerian hormone (secreted by Sertoli cells in male gonads) permits the development of Müllerian ducts to form the oviduct, uterus, and upper part of the vagina depending on the gradient of retinoic acid (RA) concentration [64,65] and region-specific *HOX* gene expressions such as *HOXA9* for the oviduct and *HOXA10/11* for the uterus [66].

The highly proliferative nature of uterine cells, especially endometrial epithelial and stromal cells, made it suitable for 3D culture approaches using primary cells obtained by endometrial biopsy for in vitro analyses of infertility-related diseases, endometriosis, and oncogenesis [67,68,69,70]. More recently, an attempt for 3D reconstitution of the uterus using the primary cells, both the epithelium and stroma, for recapitulating the human embryo implantation was reported [71]. In addition, human PSC-derived endometrium-like cells were recently derived by stepwise differentiation methods [72,73]. In addition, the innately high regenerative capacity of the uterus enables artificial reconstruction of the functional uterus for live births using biocompatible scaffolds [74].

Therefore, once we establish an appropriate in vitro reconstitution method of the uteroid in a 3D culture condition, it may serve as a robust platform applicable to infertility-related disorders, endometriosis, oncogenesis, and artificial biogenesis. Based on the close origins of the uterus/oviduct and ovary especially before invagination (Figure 4), it would be advantageous for uteroid induction to adapt the stepwise GSC differentiation method [34] combined with Müllerian duct-specific reporter systems (Supplementary Note). Slight modification of the GSC differentiation protocol from PSCs, including temporal fine-tuning of key signaling (e.g., BMP4, ActA, bFGF, WNT, and RA), may facilitate future studies on uteroids (Supplementary Note). Furthermore, using a similar approach to the uteroid, in vitro reconstitution of the oviduct (ovidutoid, named as PSC-derived oviduct-like organoid) can also serve as a platform for pathological analyses of infertility and ectopic pregnancy, since most cases of ectopic pregnancy occur in the oviduct [75].

Uterus transplantation (UTx) is now an innovative alternative to surrogacy and adoption for women with uterine factor infertility to deliver a child. To date, at least 80 UTx procedures have been performed worldwide in total, and more than 40 babies were born [76]. Although UTx has been rapidly expanding globally, it also harbors unsolved serious issues, including requirement of uterus donors, sequential surgeries such as transplantation and hysterectomy after delivery, uterine rejection and burden of immunosuppressants [77,78]. Therefore, uterine bioengineering may be considered as a remedy for future promising reproductive technology to avoid these disadvantages in UTx. Given the innately high regenerative nature of the uterus described above, PSC-derived uteroid may harbor the potential of expansion to be an actual organ size. Therefore, with the aid of biocompatible scaffolds and appropriately differentiated PSC-derived uterine cells, it is feasible to reconstitute the ominis uterus for transplantation in the future.

**Figure 5:**
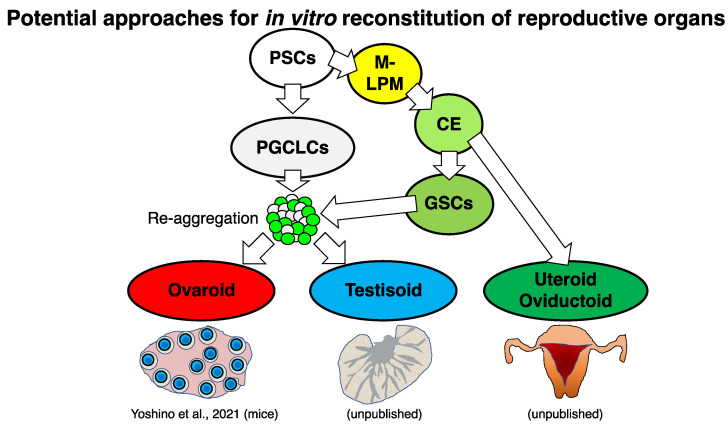
Potential approaches for in vitro reconstitution of reproductive organs. M-LPM, media- lateral plate mesoderm; CE, coelomic epithelium.

We summarize potential approaches for in vitro reconstitution of reproductive organs in Figure 5.

## 7. Ethical Concerns

Lastly, we should consider the ethical concerns of the reproductive research described above and future studies. Last year, the International Society for Stem Cell Research (ISSCR) announced the update of their guideline [79]. According to the latest guideline, although it is not achieved, the reproductive usage of gametes derived from human stem cells falls into the “Category 3A—Not allowed: Currently unsafe”. Even though researchers reported the successful in vitro gametogenesis from rodent PSCs described above, its bystander effect(s) on the next generations has not been thoroughly investigated; therefore, it requires stringent risk assessments for future clinical application. In addition, the germ-cell linage itself and reproductive organs harbor evolutional differences in multiple aspects, such as the differences in key signaling pathways and spatial cellular distributions between rodents and humans; more evolutionarily closed models are necessary to deepen our understanding of the principle and finally solve this issue. On the other hand, since the ethical consideration of uteroids for artificial reproduction that is newly proposed in this review has not been carried out, we infer that it would be required in coming years.

## 8. Conclusions

In vitro gametogenesis from rodent PSCs has opened a new era that suggests the feasibility of artificial biogenesis in large animals and ultimately in humans. Future research may transform regenerative medicine and reproduction, although ethical limitations are currently burdened on related research fields. We believe, with appropriate risk assessments and ethical consensus, that the progress of germ-cell study and attempts for in vitro reconstitution of reproductive organs would be beneficial for human beings in terms of a full grasp of primordial human origin and medical application.

## Supplementary Materials

The following supporting information can be downloaded at: https://www.mdpi.com/article/10.3390/biology11070987/s1. [34,80,81] are cited in the supplementary materials.

## Abbreviations

Pluripotent stem cells (PSCs); embryonic stem cells (ESCs); induced pluripotent stem cells (iPSCs); nonhuman primates (NHPs); gonadal somatic cells (GSCs); primordial germ cells (PGCs); primordial germ-cell-like cells (PGCLCs); spermatogonial stem cells (SSCs); spermatogonial stem cell-like cells (SSCLCs); bone morphogen protein (BMP); stem-cell factor (SCF); epidermal growth factor (EGF); leukemia inhibitory factor (LIF); epiblast-like cells (EpiLCs); media-lateral plate mesoderm (M-LPM); coelomic epithelium (CE); uterus transplantation (UTx).
