# Supplementary material for: A New Horizon in Reproductive Research with Pluripotent Stem Cells: Successful In Vitro Gametogenesis in Rodents, Its Application to Large Animals, and Future In Vitro Reconstitution of Reproductive Organs Such as “Uteroid” and “Oviductoid”
Source: Biology (Basel). 2022 Jun 29;11(7):987. doi: 10.3390/biology11070987 (PMC9312112; doi:10.3390/biology11070987)
Supplement: Supplementary file 1 [file biology-11-00987-s001.zip › biology-1769085-supplementary/MDPI_review_supple.pdf]

Supplementary Information

Supplementary Note

According to the successful GSC induction from mouse PSCs described in the previous study [1] which finally contributed to induction of functional oocytes from PSC-derived PGCLCs, GSC-related (incipient mesoderm -> media lateral plate -> coelomic epithelium -> gonad) fluorescent reporters were introduced in marmoset ESCs using a CRISPR-Cas9-mediated knock-in approach (Figure S1-S6; *T*, *OSR1*, *FOXF1*, and *GATA4*) as previously described [2]. Furthermore, additional reporter knock-in vectors were constructed for *SF1* (*NR5A1*) and *FOXL2* locus (Figure S7). Last, for the uteroid approach, knock-in reporter vectors of *Hoxa9-mTagBFP2* and *Hoxa10-mOrange2* were constructed for mouse genome (Figure S8). These vectors are available at Addgene (#186166-186175).

Supplementary Figures and legends

Figure S1

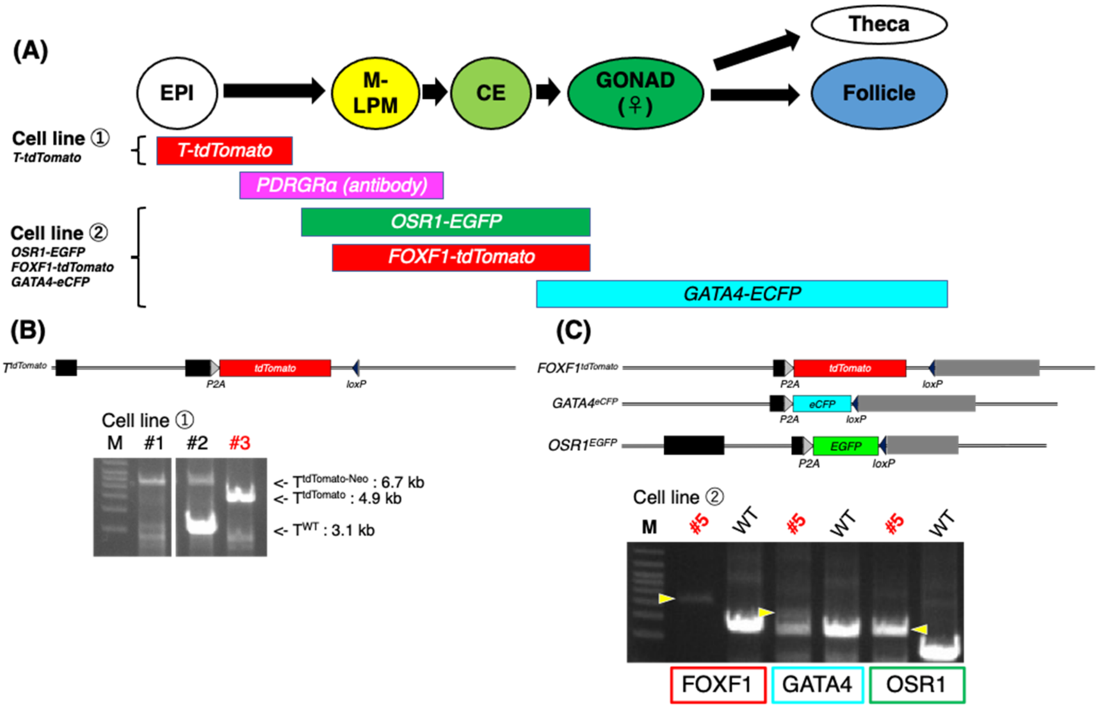

(A) Graphical schematics of the flow of GSC induction starting from PSCs. Because PDGFα is located on cell-surface, Alexa647-conjugated antibodies can be used for sorting by flow cytometry. M-LPM, media- lateral plate mesoderm; CE, coelomic epithelium. (B) Introduction of *P2A-tdTomato* into the marmoset *T* locus by knock-in. The result of genotyping PCR is shown in bottom. Used primer sequences were gacacatccaattcaaggcagac & gcactggaaaagaccctgagaat. (C) Introduction of fluorescent reporter genes into the marmoset *FOXF1*, *GATA4*, and *OSR1*. The result of genotyping PCR is shown in bottom. Knock-in bands (5.3 kb for *FOXF1*^{tdTomato}; 4.3 kb for *GATA4*^{ECFP}; 3.5 kb for *OSR1*^{EGFP}) were highlighted by yellow arrowheads. Respective wildtype (WT) bands were 3.5 kb for *FOXF1*, 3.4 kb for *GATA4*, and 2.6 kb for *OSR1*. Used primers are as follows: tctggaccctagtttgggacaat & caccagagaaattaccccctacg for *FOXF1*, ctatggtgatgtggacacgactg & gtgccaggattcttcctcagttt for *GATA4*, and tgaccaagctgtctccagaaaag & cggactcgaatttctcctcctaa for *OSR1*. Genotyping PCR and

subsequent DNA sequencing (Figures S2-S6) were performed as described previously [2]. As sgRNAs for CRISPR-Cas9-mediated knock-in, CCGTCTCAGCCGCGTTCAGC for *FOXF1*, AAAAAAGAACGAACGCTCCG for *OSR1*, ACCGCTGTGGCCTAGACGGT for *GATA4* were used.

Figure S2

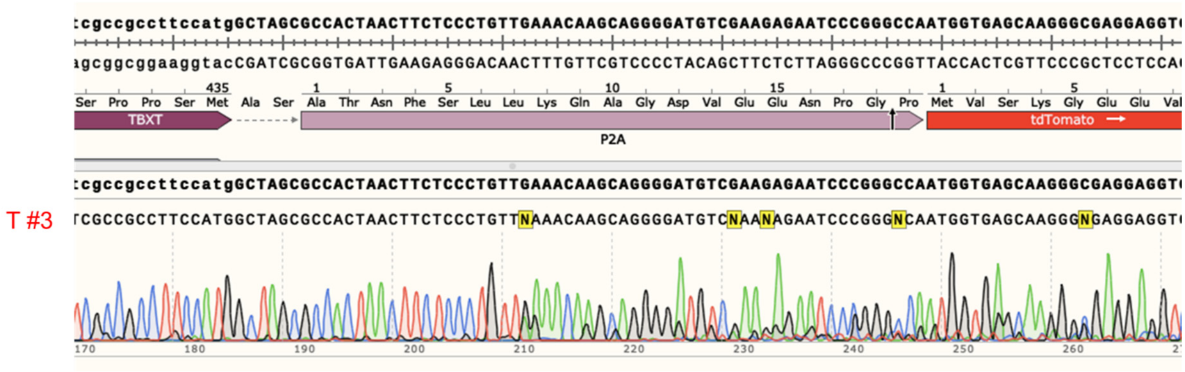

DNA sequencing for the $T^{tdtomato}$ allele in the clone #3 (Figure S1).

Figure S3

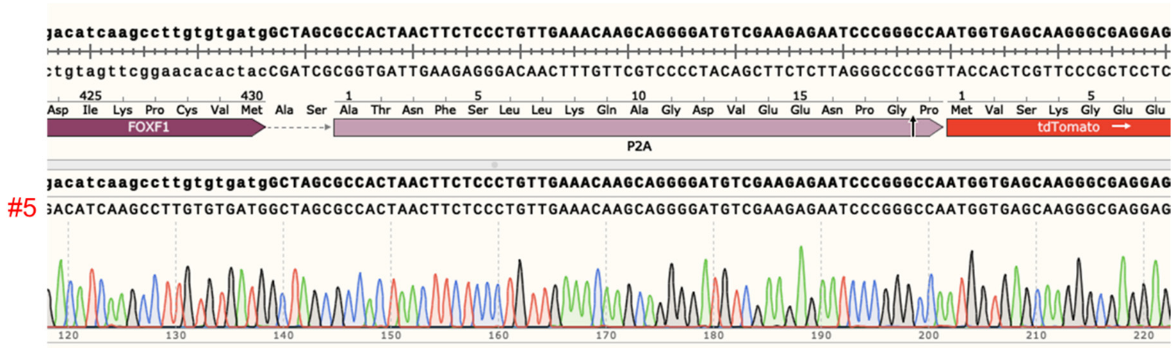

DNA sequencing for the homozygous $FOXF1^{tdtomato}$ alleles in the clone #5 (Figure S1).

Figure S4

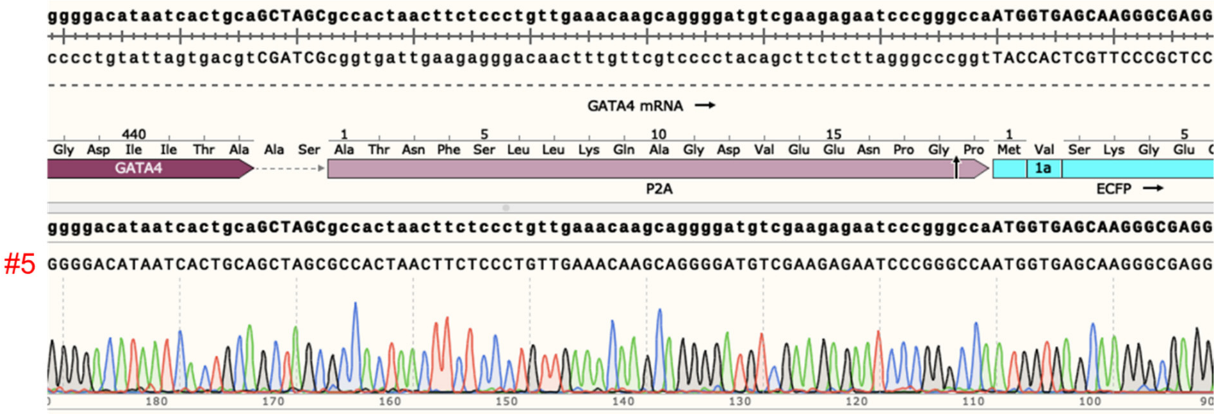

DNA sequencing for the heterozygous *GATA4*^{ECFP} allele in the clone #5 (Figure S1).

Figure S5

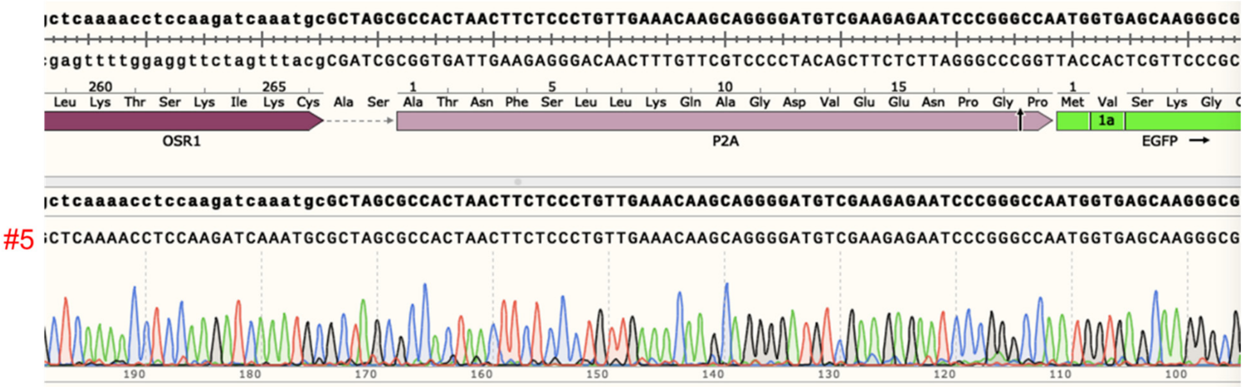

DNA sequencing for the homozygous *OSR1*^{EGFP} alleles in the clone #5 (Figure S1).

Figure S6

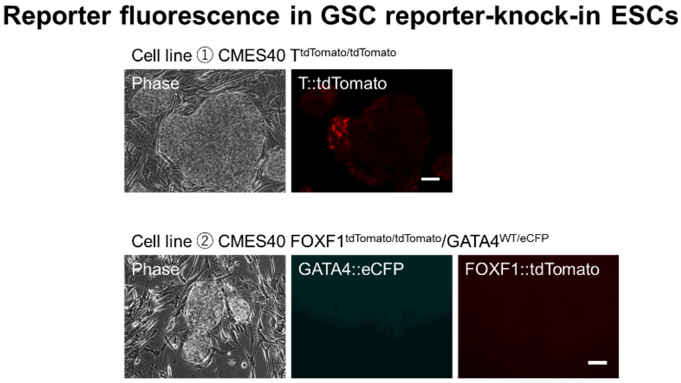

Reporter fluorescence in reporter knock-in marmoset ESCs. Only T:tdTomato ones showed fluorescent cells mosaically. Scale bars, 100 µm.

Figure S7

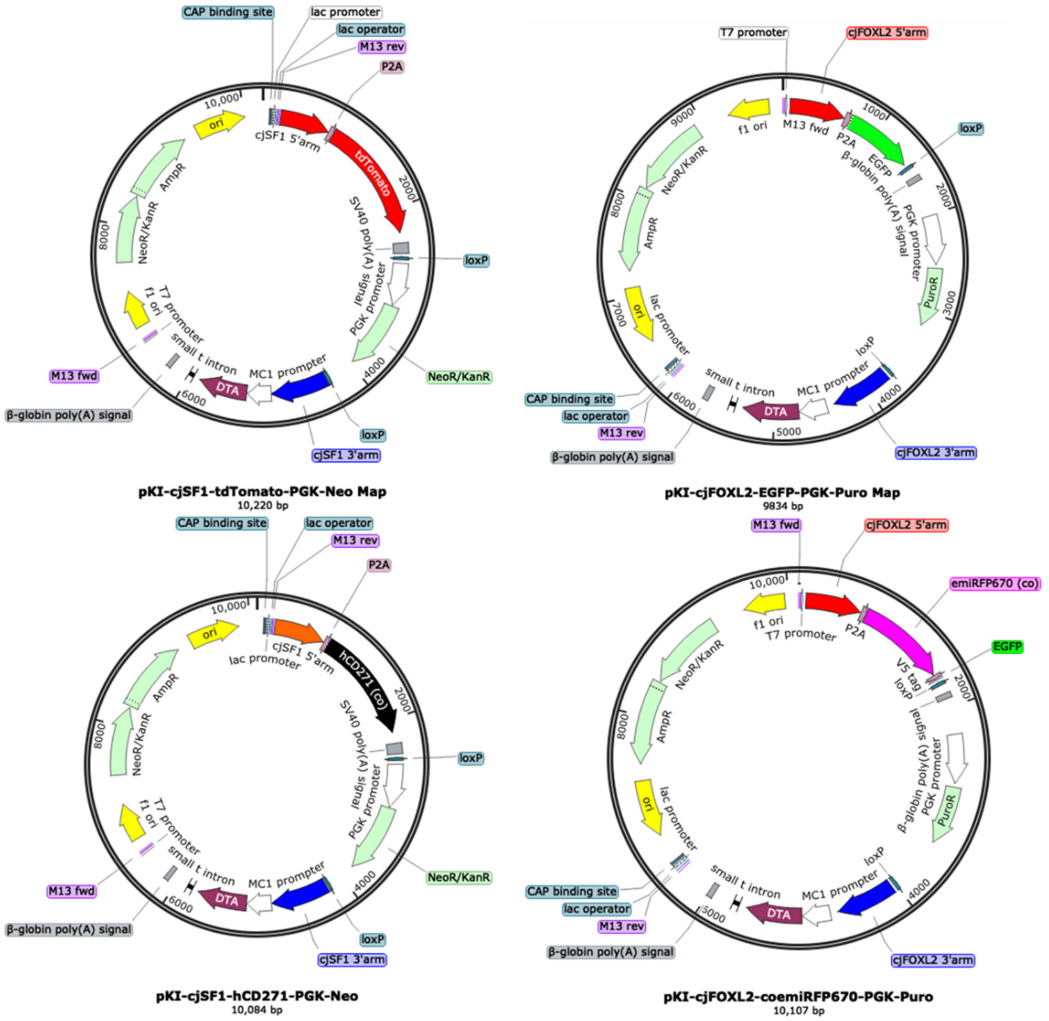

Additional reporter knock-in vectors for marmoset cells, such as *SF1-tdTomato*, *FOXL2-EGFP*, *SF1-hCD271*, and *FOXL2-coemiRFP670*. The *hCD271* and *emiRFP670* [3] sequences were human-codon-optimized (the latter was renamed *coemiRFP670*). As sgRNAs, tgcccaggctcaagtctgct for *SF1* and atcctcgcatccgtccgaac for *FOXL2* are thought to be used.

Figure S8

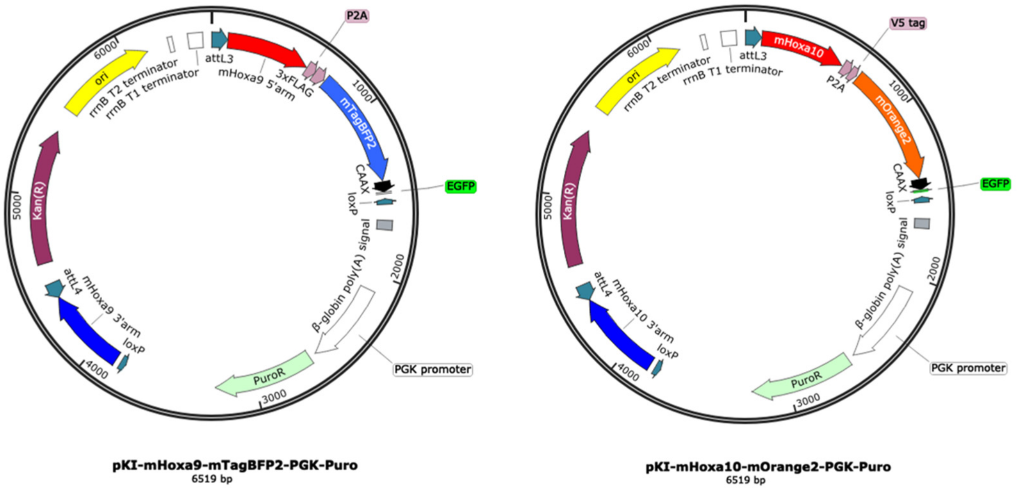

Reporter knock-in vectors of *Hoxa9-mTagBFP2* and *Hoxa10-mOrange2* for mouse cells. As sgRNAs, cccagcctccacccgcacaa for *Hoxa9* and ggaagcgaaaagacgttgtc for *Hoxa10* are thought to be used.

Supplementary References

1. Yoshino, T.; Suzuki, T.; Nagamatsu, G.; Yabukami, H.; Ikegaya, M.; Kishima, M.; Kita, H.; Imamura, T.; Nakashima, K.; Nishinakamura, R., et al. Generation of ovarian follicles from mouse pluripotent stem cells. *Science* **2021**, *373*, doi:10.1126/science.abe0237.
2. Yoshimatsu, S.; Okahara, J.; Sone, T.; Takeda, Y.; Nakamura, M.; Sasaki, E.; Kishi, N.; Shiozawa, S.; Okano, H. Robust and efficient knock-in in embryonic stem cells and early-stage embryos of the common marmoset using the CRISPR-Cas9 system. *Sci Rep* **2019**, *9*, 1528, doi:10.1038/s41598-018-37990-w.
3. Matlashov, M.E.; Shcherbakova, D.M.; Alvelid, J.; Baloban, M.; Pennacchietti, F.; Shemetov, A.A.; Testa, I.; Verkhusha, V.V. A set of monomeric near-infrared fluorescent proteins for multicolor imaging across scales. *Nat Commun* **2020**, *11*,

239, doi:10.1038/s41467-019-13897-6.
